# Frostbite Protection in Mice Expressing an Antifreeze Glycoprotein

**DOI:** 10.1371/journal.pone.0116562

**Published:** 2015-02-25

**Authors:** Martin Heisig, Sarah Mattessich, Alison Rembisz, Ali Acar, Martin Shapiro, Carmen J. Booth, Girish Neelakanta, Erol Fikrig

**Affiliations:** 1 Department of Internal Medicine, Section of Infectious Diseases, Yale University School of Medicine, New Haven, CT, United States of America; 2 Department of Infectious Disease and Clinical Microbiology, Gulhane Military Medical Academy, Haydarpasa Training Hospital, Istanbul, Turkey; 3 Section of Comparative Medicine, Yale University School of Medicine, New Haven, CT, United States of America; 4 Howard Hughes Medical Institute, Chevy Chase, MD, United States of America; University of Massachusetts Medical School, UNITED STATES

## Abstract

Ectotherms in northern latitudes are seasonally exposed to cold temperatures. To improve survival under cold stress, they use diverse mechanisms to increase temperature resistance and prevent tissue damage. The accumulation of anti-freeze proteins that improve cold hardiness occurs in diverse species including plants, arthropods, fish, and amphibians. We previously identified an *Ixodes scapularis* anti-freeze glycoprotein, named IAFGP, and demonstrated its cold protective function in the natural tick host and in a transgenic Drosophila model. Here we show, in a transgenic mouse model expressing an anti-freeze glycoprotein, that IAFGP protects mammalian cells and mice from cold shock and frostbite respectively. Transgenic skin samples showed reduced cell death upon cold storage *ex vivo* and transgenic mice demonstrated increased resistance to frostbite injury *in vivo*. IAFGP actively protects mammalian tissue from freezing, suggesting its application for the prevention of frostbite, and other diseases associated with cold exposure.

## Introduction

Ectotherms in cold environments have evolved molecular mechanisms to withstand extreme temperatures [1–5]. Very few organisms tolerate freezing solid and most avoid freezing by the accumulation of protective compounds [6,7]. Anti-freeze proteins (AFPs) contribute to cold hardiness by reducing cold-induced damage in a non-colligative manner, binding directly to the surface of ice crystals [8,9]. This interaction inhibits the addition of water molecules to the developing ice lattice and impedes crystal growth [10–12]. In turn, this prevents the formation of sharp ice needles that inflict tissue damage. These, and other adaptions, allow diverse forms of life to inhabit extreme niches, including arctic waters or glaciers, and they also contribute a survival advantage to organisms experiencing seasonal cold periods in temperate climate zones [1–5].

Shortly after the initial discovery of anti-freeze proteins by DeVries in 1969, their potential application for the preservation of foods or biological specimens was investigated [2]. The first commercial application of an anti-freeze protein, a class III AFP derived from the arctic flounder, was to reduce ice recrystallization in frozen dairy products [13–15]. The prevention of cold storage damage for clinical application has also been investigated [16–24]. Cells or tissues incubated with antifreeze proteins in hypothermic conditions demonstrated increased viability parameters when compared to controls [16,25,26]. To date only non-glycosylated AFPs have been examined in detail, as they are readily approachable by molecular techniques. Anti-freeze glycoproteins (AFGPs), despite being the initially discovered class of AFPs, are significantly less understood than other classes [9,27–29]. Their primary amino acid sequence consists of canonical Ala-Ala-Thr or Pro-Ala-Thr repeats with β-D-galactosyl-(1→3)-α-N-acetyl-D-galactosamine disaccharides attached to each threonine [27,30]. Four of these triplet repeats are sufficient for function, and up to 55 repeats have been described in arctic fish AFGPs [31]. In the black-legged tick, *Ixodes scapularis*, we recently identified an anti-freeze glycoprotein, named IAFGP [32]. *Iafgp*-expression was induced at cold temperatures and conferred a survival advantage to the tick. Fruit flies expressing *iafgp* showed enhanced cold tolerance under non-freezing hypothermic conditions [33]. Here we report that transgenic mice expressing an AFGP are resistant to frostbite.

## Results

The exposure of ectotherms to cold temperatures causes molecular adaptions that aim to prevent permanent damage and improve survival. Mammals, maintaining a fairly constant body core temperature, do not have a similar adaptive system for protection against the cold. They rely on body insulation and blood flow management to control peripheral temperatures and prevent the detrimental effects of temperature extremes. Once these measures fail, such as during extended exposure to extreme cold, local tissue damage manifests as frostbite while systemic hypothermia can lead to death [34].

**Fig 1:**
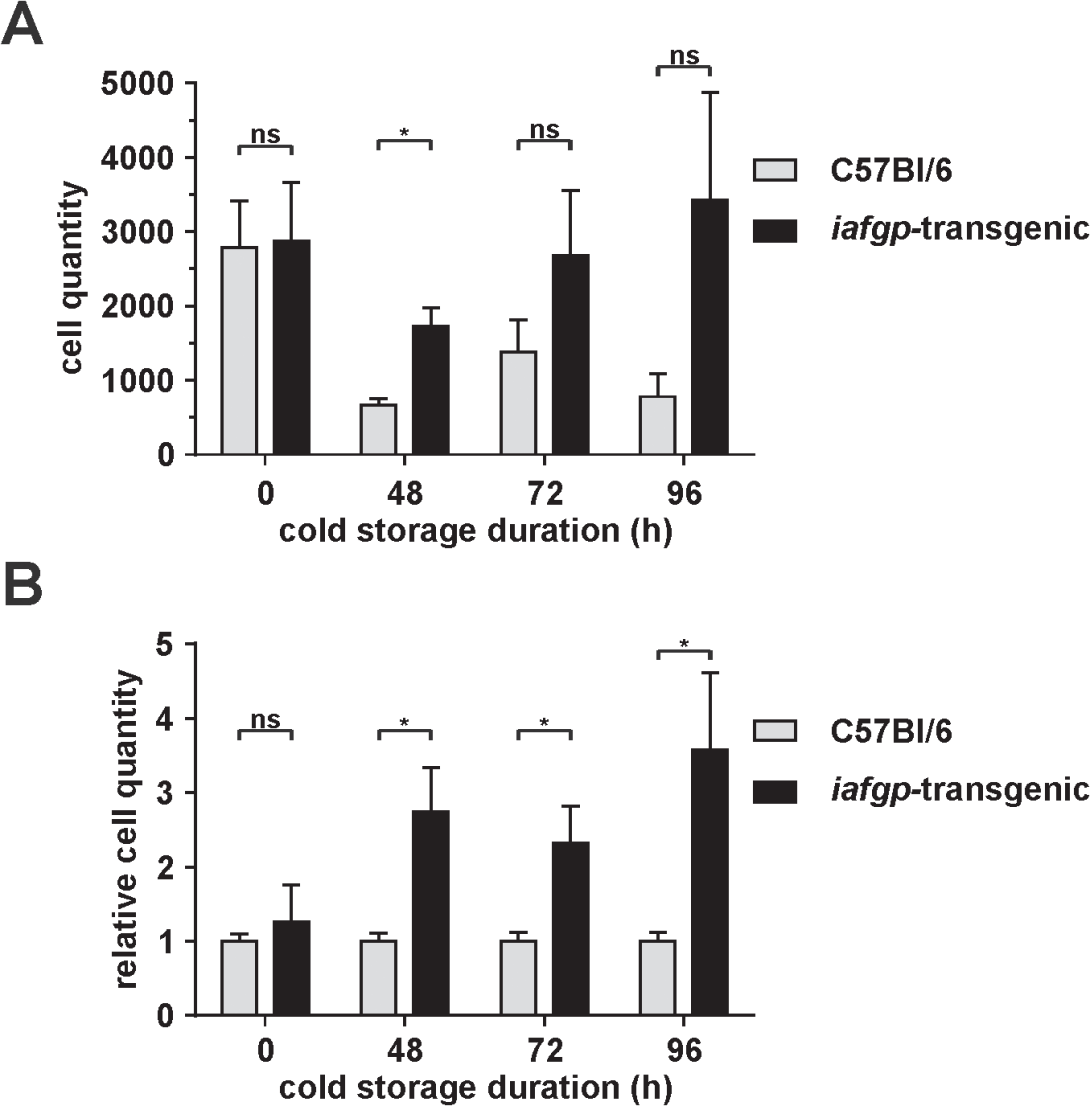
Skin fibroblasts expressing *iafgp* demonstrate increased cold resistance. Full-thickness skin punches of *iafgp*-transgenic mice and wildtype controls (C57Bl/6) were cut into small pieces and stored at 4°C for up to 4 days before shifting them to 37°C to determine fibroblast survival inside the samples. Fibroblast migration and replication was monitored microscopically and 7 days (48h cold storage) or 14 days (72h and 96 h cold storage) later the number of attached cells in each sample was quantified after extensive washing using the Guava ViaCount. A: Absolute number of recovered cells was pooled from 3–4 independent experiments with 1–2 mice per group and timepoint (*: p<0.05, Student's T-Test). The detected cell quantity comprises fibroblasts that migrated from the skin punch and cells replicating after attachment. B: The ratio of *iafgp*-transgenic cells to controls was calculated for each independent fibroblast migration experiment and pooled per timepoint (*: p<0.05, Student's T-Test).

To investigate cold protection of mammalian cells and tissues by an antifreeze glycoprotein, we recently generated a transgenic mouse constitutively expressing *iafgp* [35]. Skin fibroblast survival during cold storage was compared between *iafgp*-transgenic mice and controls *ex vivo*. Full-thickness skin punches were cut into pieces and stored at a liquid-air interface at 4°C for up to 4 days, before shifting them to 37°C to determine fibroblast survival inside the samples. Fibroblast migration and replication were monitored microscopically and cell titers were quantified up to 14 days after cold storage by flow cytometry using the Guava ViaCount. Skin punch samples from transgenic animals demonstrated improved cell survival after hypothermic incubation, while untreated control samples showed no difference in fibroblast migration (Fig. 1A; 2 day cold storage: p<0.05; 3, 4 day cold storage: non-significant). In contrast to untreated samples, the relative cell counts were increased 2–4 fold following cold storage at 4°C (Fig. 1B).

**Fig 2:**
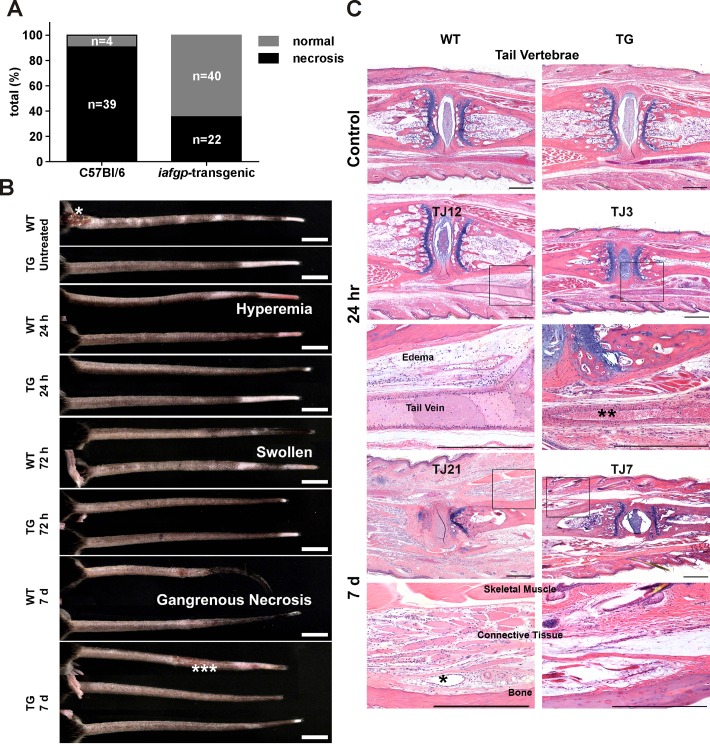
*Iafgp*-transgenic mice are resistant to frostbite. The tails of homozygous *iafgp*-transgenic mice were immersed into a -22°C liquid for 4 minutes. Tissue autoamputation following frostbite was determined 7 to 10 days later. A: Shown data were pooled from 9 independent experiments comprising up to two batches (n corresponds to the number of animals; Fisher's exact test, ***: p<0.0001). B: Representative tail images from wildtype (WT) and *iafgp*-transgenic (TG) mice at 24 hours, 72 hours and 7 days post thermal injury inflicted on the distal 5 cm of the murine tails. Tail injury from fighting (*) occurred sporadically at the tail base and these sites were excluded from evaluation. About 35% of the transgenic mice showed macroscopic changes consistent with thermal injury (***) but less severe than control mice. Scale bars = 1 cm. C: Representative histopathology images of tails from untreated controls,wildtype (WT) and *iafgp*-transgenic (TG) mice at 24 hours and 7 days post thermal injury. Complete loss of nuclei in skeletal muscle and bone with empty blood vessels (*) in the intervening connective tissue in WT tails and viable tissue or red blood cells in TG tails can be observed (**). TJ = tail joint numbered starting from the most distal joint. Tails are oriented proximal left and distal right. Scale bars = 500 μm.

The *iafgp*-transgenic mice were examined using an *in vivo* frostbite model to extend the studies to a situation that is clinically relevant for human. Tails of anesthetized mice were immersed in a -22°C cold bath and in the ensuing days, the progressing tissue necrosis was assessed and carefully demarcated. While only 11% of the control mice remained free of frostbite during the follow-up period, 60% of the transgenic animals showed no visible signs of frostbite injury (Fig. 2A, Fisher’s exact test, p<0.0001). In this model the frostbite injury leads to visible necrosis of tail tissue (Fig. 2B). At 24 hours, wild-type (WT) mice tails were visibly red (hyperemic) in contrast to *iafgp*-transgenic (TG) mice that appeared grossly normal. By 72 hours WT tails were markedly swollen and cold to the touch when compared to TG mice that were mostly normal. By day 7, all WT tails were shrunken, dry, and black—consistent with the gangrenous form of coagulation necrosis that is often correlating with ischemic injuries. In contrast, at this time point TG mice were mostly normal and only a limited number had minor macroscopic changes consistent with thermal injury—which was much less severe than WT mice. Tail histopathology confirmed the increased severity of edema and necrosis in WT animals in comparison to TG mice (Fig. 2C). Untreated tails showed normal tissue morphology for bone, skin, connective tissue, and blood vessels in TG and WT mice. 24 hours post thermal injury, WT mice demonstrated visible changes of acute coagulation necrosis with distention of the tail vein by clotted blood and perivascular edema. In contrast, transgenic tails were viable with minimal to no changes, including normal red blood cells. By day 7 post thermal injury, WT mice manifested diffuse coagulation necrosis with complete loss of nuclei in skeletal muscle and bone, and blood vessels lacking red blood cells in the intervening connective tissue. Similar morphologic changes were observed in TG mice at day 7, however, they occurred further distal to the changes observed in WT mice (Fig. 2C).

**Fig 3:**
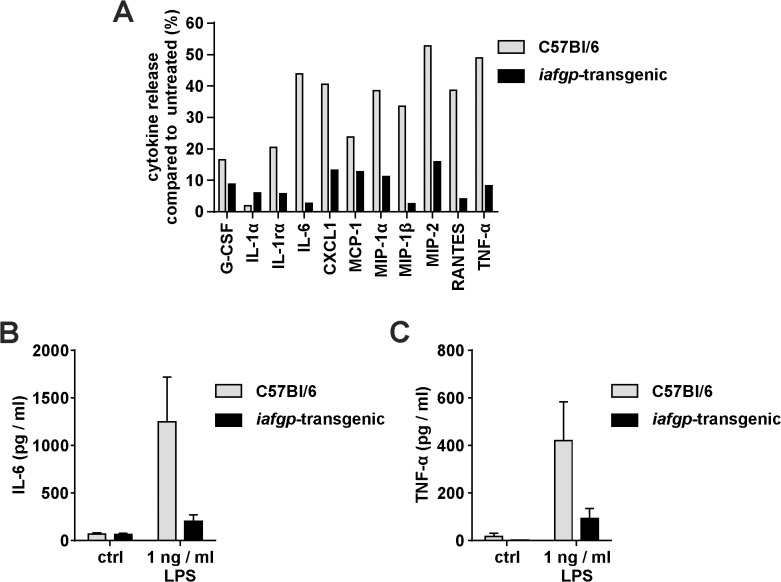
*Iafgp*-expression reduces inflammation. Peritoneal macrophages (PMO) isolated from *iafgp*-transgenic mice and controls were stimulated *ex vivo* with *E. coli* LPS and cytokine production was quantified. A: *In silico* quantified cytokine array intensities of samples stimulated with 1 ng/ml LPS in comparison to unstimulated controls are shown for C57Bl/6 control mice and transgenic animals. The corresponding cytokine array raw data is depicted in S1 Fig. Overall cytokine release is significantly reduced between the two mouse lines (Two-Way-ANOVA; p = 0.003). This experiment was performed once. B: IL-6 - or C: TNF-α release determined by ELISA using PMOs stimulated with 1 ng/ml LPS. Shown data represents the average TNF-α concentration of three independent stimulations ± SEM.

In addition to the direct tissue injury caused by frostbite, the inflammatory immune response contributes to pathologic changes. The immune response to a sterile injury, similar to the response against pathogens, is caused by invasion of immune cells and the secretion of inflammatory cytokines [36]. To investigate the influence of IAFGP on the inflammatory response to frostbite, IL-6 and TNF-α serum cytokine levels were measured by ELISA in TG and WT mice. The cytokine titers remained below the detection limit, likely due to the local nature of the tail freeze procedure (data not shown). *Ex vivo* stimulation of *iafgp*-transgenic peritoneal macrophages, however, with 1 ng / ml LPS released considerably lower levels of G-CSF, IL1-rα, IL-6, CXCL1, MCP-1, MIP-1α, MIP-1β, MIP-2, RANTES and TNF-α than control cells (Fig. 3A; corresponding raw data in S1 Fig.). The induction of inflammatory cytokines following LPS stimulation was 2-fold to 16-fold lower in TG cells than in WT cells. Using ELISA to quantify the IL-6 or TNF-α release in response to LPS stimulation, the diminished inflammatory response of TG cells was confirmed (Fig. 3B, C).

## Discussion

Mammals maintain their body temperature, under diverse environmental conditions, within a small thermal range. Local contact with extreme temperatures causes burn wounds or frostbite, and systemic exposure to extreme cold or heat can lead to death [34]. Organisms without intrinsic temperature control have adapted to thrive within a wide range of body temperatures using various mechanisms to prevent harmful effects [1–5]. One strategy to survive cold temperatures, the expression of AFPs, prevents cold damage by modifying ice formation. These proteins bind to the surface of small ice nuclei and alter ice crystal growth, shape or size. AFPs are abundant in diverse organisms encountering cold or freezing environments, including fish, arthropods, and plants. One subgroup of the structurally diverse family of antifreeze proteins, the antifreeze glycoproteins (AFGP), is still poorly understood, in part because of its challenging molecular properties, which have complicated effective recombinant protein expression and purification [9]. Highly repetitive primary DNA and amino acid sequences, pronounced glycosylation, and the proposed degradation into functional peptides thwart the application of standard techniques.

We recently discovered the first AFGP in the black-legged tick, *I. scapularis*, and demonstrated that this protein, IAFGP, is sufficient to confer cold resistance to transgenic arthropods [32,33]. To investigate IAFGP function in a mammalian model system, we generated a transgenic mouse expressing IAFGP. Transgene expression was confirmed in all examined murine tissues throughout multiple breeding generations. Protection from cold shock was investigated *ex vivo* following hypothermic incubation of skin biopsies. *Iafgp*-transgenic mouse samples showed earlier and higher fibroblast migration compared to controls, indicating increased tolerance to the cold. Most importantly, *in vivo* the freezing resistance of the *iafgp*-transgenic mouse was examined in a frostbite model. Frostbite, one of the most dangerous forms of local cold, is caused by the intercellular and intracellular formation of ice crystals that rupture cellular membranes, leading to uncontrolled cell death. The release of cytosolic content then results in leukocyte recruitment, swelling and tissue inflammation [36]. In addition to the initial destruction caused by frostbite, the subsequent inflammatory reaction during tissue reperfusion contributes to the permanent damage that is evident after frostbite [37,38].

We hypothesized that *iafgp*-expression decreases the damage during frostbite, thereby diminishing the inflammatory cascade associated with cell death. Indeed, mouse tails of *iafgp*-transgenic mice were less likely to freeze in subzero conditions than controls (38% versus 93% respectively). In tails that remained unfrozen the tissue injury was repaired over time without permanent damage, while frozen tails progressed to autoamputation. The higher rate of autoamputation among controls (91% versus 35% in transgenic mice) therefore corresponds to the higher number of frozen versus unfrozen tails. There seems to be a threshold of cold damage that tissues can withstand without progressing to lasting autoamputation [39]. In our model IAFGP prevented tail freezing, therefore reducing direct tissue damage and the inflammation that leads to lasting destruction. Our data also demonstrate a pronounced anti-inflammatory effect of IAFGP during LPS stimulation, suggesting a potential beneficial function not only in damage prevention but also after frostbite damage occurred. Indeed histopathologic analysis of the few *iafgp*-transgenic tail samples that showed frostbite damage revealed reduced injury when compared to the control samples.

Our data demonstrate that IAFGP affords protection from frostbite in a transgenic mouse line—the first *in vivo* demonstration of the functional influence of an antifreeze-glycoprotein expressed in an adult mammal. Investigating its function in a mammalian system *in vivo* without the need for recombinant expression or characterization of the glycosylation status, is a versatile approach that can be applied to other anti-freeze glycoproteins. Numerous medical conditions where cold exposure is deleterious including frostbite and Raynaud’s syndrome [40] can be assessed in greater detail. In addition, these proteins may show promise in transplantation where hypothermic damage during ischemic storage can influence organ viability. The anti-inflammatory function could be of additional benefit during organ reperfusion after implant. AFGPs may also synergize with the recently described approach of supercooled organ storage, that elegantly extends organ preservation by sub-zero storage without freezing [41]. All of these processes, and the mechanisms by which protection is conferred, can now be examined in IAFGP-expressing mice. These efforts may lead to the use of IAFGP, and possible other antifreeze glycoproteins, in various medical applications.

## Experimental procedures

### Characterization of the *iafgp*-expressing mouse line

*Iafgp* was constitutively expressed under control of the chicken β-*actin* promoter in an *iafgp*-transgenic mouse line [35]. Transgenic *iafgp*-expressing mice were backcrossed for 8 generations to C57Bl/6 mice and bred to homozygosity (Charles River, MA). Experimental mice of 6–12 weeks were housed at 20–22°C with water and food *ad libitum*. Animal handling was performed according to protocols approved by the Yale animal care and use committee.

To examine the murine frostbite response, homozygous *iafgp*-transgenic mice were anesthetized using Ketamine/Xylazine (100 mg / 10 mg per kg) and the distal end of their tails was immersed into a 22°C liquid bath. For the histopathology experiment the distal 5 cm of the tails were immersed. For other tail freeze experiments the tail was immersed except for the proximal 2 cm of tail. Each experimental batch comprised a mix of up to 10 wild-type and transgenic mice. Following the procedure mice were transferred back into their cages for recovery and provided with Metacam-supplemented (0.5 μg/ml) water for analgesia *ad libitum*. Tissue necrosis following frostbite challenge was determined visually 7 to 10 days later. Control animals were age and sex-matched to transgenic animals, animal weights between groups showed no significant difference (data not shown). Animal handling was performed according to protocols approved by the Yale animal care and use committee. Mice were kept in groups under a 12h light cycle with food and water *ad libitum*. Tissue histology was analyzed in Hematoxylin/Eosin-stained sections of decalcified (Decalcifier, Electron Microscopy Sciences, Hatfield, PA) tail samples fixed in Bouin’s solution (Ricca Chemical Co., Arlington, TX). Mice used for histopathologic examination were examined with reviewer (CJB) blinded to experimental genotype or manipulation.

### Fibroblast migration assay and stimulation of peritoneal macrophages

*Iafgp*-transgenic mice and matched C57Bl/6 controls were euthanized and the abdomen was shaved. Following alternating disinfection with ethanol wipes and iodine surgical scrub, whole-thickness skin was removed and transferred into DMEM supplemented with 10% FCS. Skin punches of 3.5 mm diameter transferred into 24-well microtiter plates were cut into small pieces and stored for 2–4 days at 4–6°C before being shifted to 37°C for up to 14 days. Fibroblast migration was monitored using a microscope and 7 days (48h cold storage) or 14 days (72h and 96 h cold storage) later the final cell counts were determined. Following extensive washing with PBS to remove non-adherent tissue pieces and release of fibroblasts using trypsin the total cell quantity was measured using flow cytometry (Guava ViaCount, EMD Millipore, Darmstadt, Germany) according to manufacturer’s recommendations.

Peritoneal macrophages were isolated from matched 6–10 week old mice according to standard protocols [42]. 1 x 10^5^ cells were seeded per well in microtiter plates and incubated for 16h at 37°C with 5% CO_2_ before stimulation with 1, 5, 10 or 100 ng/ml LPS isolated from *E. coli* 0111:B4 (Sigma, MO). 24h post stimulation the supernatants were removed and cytokine levels were analyzed using ELISA or mouse cytokine array (R&D Systems, MN).

### Statistical analysis

All experiments were repeated independently at least 3 times, if not noted otherwise. Statistical differences between groups were analyzed using Students T-Test and Fishers exact test. Analyses were performed using two-sided tests. P values below 0.05 were considered significant and marked with one asterisk. Calculations were performed using Excel 2007 (Microsoft, WA), graphing and statistics were performed using Prism 6.0 software (GraphPad Software Inc., CA).

## Supporting Information

S1 Fig Iafgp-expression reduces inflammation. Peritoneal macrophages (PMO) isolated from C57Bl/6 wildtype mice and transgenic animals were stimulated for 24h with 1 ng/ml E. coli 0111:B4 LPS (Sigma, MO). Cytokine release into the cell supernatants was assessed using an murine cytokine array (R&D Systems, MN). Depicted are the scanned images of four arrays incubated with LPS treated or untreated cells derived from wildtype or transgenic animals. Spot intensities correlate with cytokine quantities. In silico quantified data is shown in Fig. 3A.
(PDF)
