# Supplementary material for: Frostbite Protection in Mice Expressing an Antifreeze Glycoprotein
Source: PLoS One. 2015 Feb 25;10(2):e0116562. doi: 10.1371/journal.pone.0116562 (PMC4340617; doi:10.1371/journal.pone.0116562)
Supplement: S1 Fig — Peritoneal macrophages (PMO) isolated from C57Bl/6 wildtype mice and transgenic animals were stimulated for 24h with 1 ng/ml E. coli 0111:B4 LPS (Sigma, MO). Cytokine release into the cell supernatants was assessed using an murine cytokine array (R&D Systems, MN). Depicted are the scanned images of four arrays incubated with LPS treated or untreated cells derived from wildtype or transgenic animals. Spot intensities correlate with cytokine quantities. In silico quantified data is shown in Fig. 3A. (PDF) [file pone.0116562.s001.pdf]

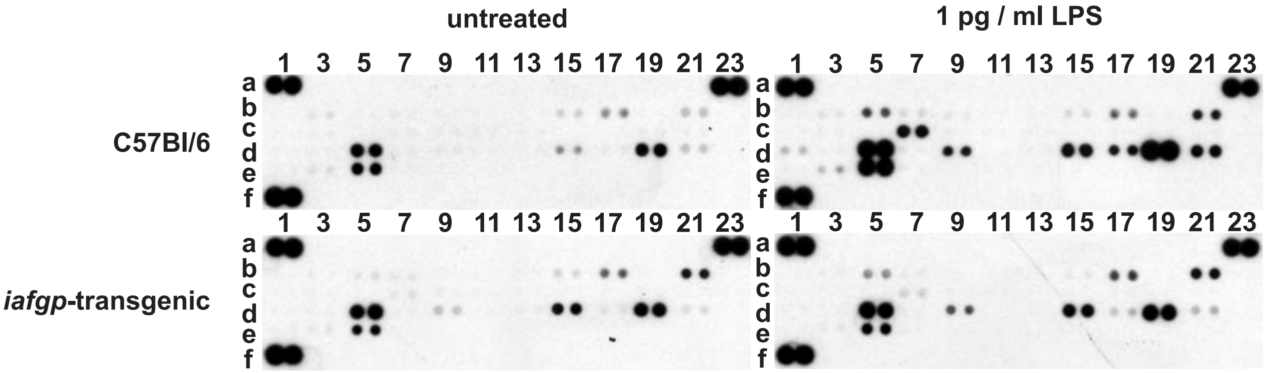

b1	BLC, CXCL13, BCA-1	c21	IL-23
b3	C5/C5a Complement Comp 5a	c23	IL-27
b5	G-CSF	d1	IP-10, CXCL10, CRG-2
b7	GM-CSF	d3	I-TAC, CXCL11
b9	I-309, CCL1, TCA-3	d5	KC, CXCL1
b11	Eotaxin, CCL11	d7	M-CSF
b13	sICAM-1, CD54	d9	JE, CCL2, MCP-1
b15	IFN-γ	d11	MCP-5, CCL12
b17	IL-1α, IL-1F1	d13	MIG, CXCL9
b19	IL-1β, IL-1F2	d15	MIP-1α, CCL3
b21	IL-1rα, IL-1F3	d17	MIP-1β, CCL4
b23	IL-2	d19	MIP-2, CXCL2
c1	IL-3	d21	RANTES, CCL5
c3	IL-4	d23	SDF-1, CXCL12
c5	IL-5	e1	TARC, CCL17
c7	IL-6	e3	TIMP-1
c9	IL-7	e5	TNF-α
c11	IL-10	e7	TREM-1
c13	IL-13		
c15	IL-12 p70	a1	assay pos ctrl
c17	IL-16	a23	assay pos ctrl
c19	IL-17	f1	assay pos ctrl

Supplementary figure 1. *Iafgp*-expression reduces inflammation.
Peritoneal macrophages (PMO) isolated from C57Bl/6 wildtype mice and *iafgp*-transgenic animals were stimulated for 24h with 1 ng/ml E. coli 0111:B4 LPS (Sigma, MO). Cytokine release into the cell supernatants was assessed using an murine cytokine array (R&D Systems, MN). Depicted are the scanned images of four arrays incubated with LPS treated or untreated cells derived from wildtype or transgenic animals. Spot intensities correlate with cytokine quantities.
In silico quantified data is shown in Figure 3a.

Supplementary figure 1
